# Upregulation of Mitochondrial Content in Cytochrome *c* Oxidase Deficient Fibroblasts

**DOI:** 10.1371/journal.pone.0165417

**Published:** 2016-10-25

**Authors:** Aviram Kogot-Levin, Ann Saada, Gil Leibowitz, Devorah Soiferman, Liza Douiev, Itamar Raz, Sarah Weksler-Zangen

**Affiliations:** 1 The Diabetes Unit, Department of Internal Medicine, Hadassah-Hebrew University Medical Center, Jerusalem, Israel; 2 Monique and Jacques Roboh Department of Genetic Research and Department of Genetic and Metabolic Diseases, Hadassah-Hebrew University Medical Center, Jerusalem, Israel; Universidad Pablo de Olavide, SPAIN

## Abstract

Cytochrome-c-oxidase (COX) deficiency is a frequent cause of mitochondrial disease and is associated with a wide spectrum of clinical phenotypes. We studied mitochondrial function and biogenesis in fibroblasts derived from the Cohen (CDs) rat, an animal model of COX deficiency. COX activity in CDs-fibroblasts was 50% reduced compared to control rat fibroblasts (P<0.01). ROS-production in CDs fibroblasts increased, along with marked mitochondrial fragmentation and decreased mitochondrial membrane-potential, indicating mitochondrial dysfunction. Surprisingly, cellular ATP content, oxygen consumption rate (OCR) and the extracellular acidification rate (ECAR) were unchanged. To clarify the discrepancy between mitochondrial dysfunction and ATP production, we studied mitochondrial biogenesis and turnover. The content of mitochondria was higher in CDs-fibroblasts. Consistently, AMPK activity and the expression of NRF1-target genes, NRF2 and PGC1-α that mediate mitochondrial biogenesis were increased (P<0.01 vs control fibroblast). In CDs-fibrobalsts, the number of autophagosomes (LC3+ puncta) containing mitochondria in CDs fibroblasts was similar to that in control fibroblasts, suggesting that mitophagy was intact. Altogether, our findings demonstrate that mitochondrial dysfunction and oxidative stress are associated with an increase in mitochondrial biogenesis, resulting in preservation of ATP generation.

## Introduction

Cytochrome *c* oxidase (COX, complex IV) is the last enzyme of the mitochondrial respiratory chain and is a regulation site for mitochondrial oxidative phosphorylation (OXPHOS) system. COX catalyzes the transfer of electrons from reduced cytochrome *c* to molecular oxygen [1,2] in a reaction coupled to proton pumping across the inner mitochondrial-membrane, thereby assisting the establishment of the mitochondrial electrochemical gradient [3].

COX deficiency in humans is a commonly recognized cause of mitochondrial disorder [4]. In addition, alteration of COX function was demonstrated in a diverse array of pathological conditions such as Alzheimer’s and Parkinson’s diseases, and age related loss of COX activity was also reported [5,6]. For diagnostic testing, cultured skin fibroblasts are an attractive tissue to evaluate OXPHOS function because of the minimally invasive character of sampling and the amount of cell material obtained by culturing [7]. Studies in human COX-deficient fibroblasts, derived from patients, have revealed diminished COX activity, compromised ATP production, reactive oxygen species (ROS) overproduction and abnormal mitochondrial morphology [8–10].

The Cohen rat (CDs) is an inbred genetic model of COX deficiency [11–13] exhibiting partially reduced COX activity in different tissues. The CDs rats demonstrate no overt pathology but presents reduced pregnancy and fertility rate, increased embryonic mortality and lower body weight [14]. Previous studies in the CDs model have shown that a severe COX impairment in pancreatic-islets induced by high-sucrose low-copper diet was associated with diabetes development [11,13]. We recently showed that islets isolated from CDs rats exhibited a consistent partial reduction in COX activity, ATP content and glucose stimulated insulin secretion, which could be recovered by feeding on a copper-sufficient diabetogenic-diet. These observations are supporting the involvement of COX in β-cell dysfunction in CDs rat islets [12].

Since COX-deficiency is ubiquitous, primary fibroblasts provide a useful tool to study the CDs mitochondrial phenotype. In this study, we examine the association between COX-deficiency and mitochondrial biogenesis in the CDs-fibroblasts.

## Methods

### Cell culture

Fibroblasts were obtained from rat ear biopsies of CDs and control Cohen diabetes-resistant (CDr) rats fed regular chow-diet (Teklad, Harlan Laboratories, USA). The control CDr strain is genetically derived experimental model that maintains normoglycemia on the regular diet and the diabetogenic high sucrose low copper diet [11]. Fibroblasts were cultured in DMEM medium containing glucose (4.5 g/L), 10% fetal calf serum (FCS) and 100 IU/mL penicillin/streptomycin (DMEM-Glu) (All from Biological Industries, Kibbutz Beit Haemek, Israel) at 37°C, 5% CO_2_. The DMEM-Glu culture medium was used for all experiments unless otherwise stated. To induce starvation, fibroblasts were washed with phosphate-buffered saline (PBS) and incubated in glucose-free DMEM without fetal calf serum. For autophagy analysis, cells were incubated with or without 100 nM of bafilomycin A1 for 4 h.

### Enzymatic activities of cytochrome *c* oxidase and citrate synthase

Enzymatic activities of COX and of citrate synthase (CS), a mitochondrial control enzyme, were determined in cell or tissue homogenates by standard spectrophotometric methods as previously described [15]. Briefly, COX activity was measured by following the oxidation of reduced cytochrome *c* at 550 nm. CS activity was measured in the presence of acetyl-CoA and oxaloacetate by monitoring the release of CoASH coupled to 5′5-dithiobis (2-nitrobenzoic) acid at 412 nm. COX activity was expressed as a ratio normalized to that of CS.

### Assays

For the different assays 2000 cells/well were seeded in quadruplicate on four 96-well cell culture plates as we have previously described [16]. The following day, medium was removed and replaced with 100 μl of fresh DMEM-Glu permissive medium or a restrictive glucose-free medium (Biological Industries, Kibbutz Beit Haemek, Israel) supplemented with 10% dialyzed FCS, 5 mM galactose, 2 mM l-glutamine and 100 IU/mL penicillin/streptomycin (DMEM-Gal). Following 72 h, tissue cultures were analyzed for cell biomass, ROS, ATP, membrane potential and mitochondrial content.

Cell biomass was measured by a colorimetric method using methylene blue (MB) as described [16]. Cells were fixed with 0.5% glutaraldehyde for 10 min, rinsed with water, stained with 1% MB in 0.1 M borate buffer for 1 h, rinsed with water and allowed to dry. The dye was extracted from the cells using 0.1 N HCl at 37°C for 1 h and then measured at A_620_ nm.

Intracellular ROS production was measured using 2′,7′-dichlorodihydrofluorescein diacetate (DCF) (Biotium, Harvard, CA, USA) [17]. Briefly, growth medium was removed and replaced with 100 μl of 10 μM DCF in PBS-Ca^2+^ Mg^2+^ (PBS containing 0.9 mM CaCl_2_ and 0.5 mM MgCl_2_) and incubated for 25 min at 37°C, 5% CO2. DCF was replaced with 100 μl PBS-Ca^2+^ Mg^2+^ and ROS production was monitored for 20 min at λex 485 nm, λem 520 nm.

ATP level was quantified by the ATPlite^®^ luciferin-luciferase bioluminescence assay according to the manufacturer's instructions (Perkin Elmer Waltham MA, USA).

Mitochondrial content and mitochondrial membrane potential were estimated, respectively, using MitoTracker Green FM (MTG) and tetramethylrhodamine ethyl ester (TMRE) (Molecular Probes, Eugene, OR, USA). MitoTracker Green was added to the existing medium to a final concentration of 200 nM and cells were incubated for 45 min at 37°C, 5% CO_2_. TMRE was added successively to a final concentration of 50 nM and the cells were incubated for an additional 45 min at 37°C, 5% CO_2_. Medium was removed and after rinsing once with PBS, replaced with 100 μl PBS. The plate was read at 37°C, *λ*_ex_ 485 nm, *λ*_em_ 528 nm (MTG) and *λ*_ex_ 485 nm, *λ*_em_ 590 nm (TMRE).

Absorbance, fluorescence and luminescence measurements were performed using a Synergy HT microplate reader (Bio-Tek Instruments, Vinoosky, VT, USA) at 30°C unless otherwise specified. Relative fluorescence units (RFU) and relative luminescence units (RLU) were calculated by normalizing to cell biomass as measured by MB.

### Fluorescence microscopy

Fibroblasts were grown in DMEM-Glu and stained with 250 nM MitoTracker Red CMXRos (Invitrogen). Cells were examined with a confocal microscope (40×/100×) (Olympus BX-UCB). All micrographs were taken under the same conditions and the fluorescence intensity of the mitochondria relative to the cell volume was calculated in 200 cells using the ImageJ software (NIH, Bethesda, MD).

### Immunofluorescence staining

Fibroblasts grown on glass coverslips were incubated with 2 μM MitoTracker Red CMXRos (Invitrogen) for 45 min at 37°C, 5% CO2. Cells were washed in PBS and fixed with 4% paraformaldehyde for 15 min and then permeabilized for 10 min with 0.3% Triton X-100. Following, blocking was performed with 2.5% bovine serum albumin in PBS for 30 min. Subsequently, cells were incubated with primary antibodies directed against LC3B (microtubule-associated protein 1 light chain-3B, Cell signaling) for 2 h at room temperature. After washing, they were incubated for 1 h at room temperature with Cy3-conjugated anti-rabbit secondary antibodies (Jackson ImmunoResearch Laboratories, West Grove, PA), washed, mounted on slides and examined with a confocal microscope (Olympus BX-UCB).

### XF24 Seahorse Flux Analyzer

Oxygen consumption rate (OCR) and extra-cellular acidification rate (ECAR) were measured using an XF24 extracellular flux analyzer (Seahorse Biosciences). Fibroblasts were seeded in an XF 24-well cell culture plate (Seahorse Bioscience) at a density of 20×10^3^ cells/well in 300 μl of DMEM-Glu medium and incubated for 24h at 37°C, 5% CO_2_. The following day the medium was replaced with fresh DMEM-Glu. After 48hrs, the growth medium was changed to 500 μl of unbuffered DMEM, pH 7.4, and cells were incubated at 37°C without CO_2_ for 1h for equilibration before starting the assay. OCR and ECAR were measured simultaneously for ~3 min in repeated cycles, to obtain basal rates. After baseline measurements, OCR and maximal ECAR were measured after the injection of Oligomycin to a final concentration of 1 μM. Subsequently, Carbonyl cyanide-4-(trifluoromethoxy)phenylhydrazone (FCCP) was injected to reach a working concentration of 5 μM and the maximal OCR was measured. Non-mitochondrial oxygen consumption was measured after the injection of rotenone and Antimycin A to a final concentration of 3 μM each. All OCR and ECAR values were normalized to cell biomass measured by the methylene blue method.

### Western blot analysis

Protein samples were electrophoresed on a 12% SDS-PAGE and transferred to a nitrocellulose-membrane. Blot was blocked and incubated with anti-COX1 (MitoSciences), anti-phospho-AMPKα-(Thr^172^) (phosphorylated adenosine monophosphate activated protein kinase, Cell signaling), phospho-ACC-(Ser^79^) (phospho-acetyl-CoA carboxylase, Cell signaling), PGC-1α (Peroxisome proliferator-activated receptor gamma coactivator 1-alpha, Abcam) or anti-LC3B (Cell signaling) antibodies overnight at 4°C. Membrane was washed and incubated with horseradish peroxidase-conjugated secondary antibody for 1 h at room temperature and bound antibodies were detected using the SuperSignal West Pico chemiluminescent substrate (Thermo Scientific, USA). Band densities were analyzed using the ImageJ software.

### Real-time PCR

Total RNA was isolated from fibroblasts using Tri-Reagent (Telron, Israel). First-strand cDNA synthesis was performed using the High Capacity cDNA Reverse Transcription kit (Applied Biosystems). Real-time PCR (RT-PCR) was performed using Fast SYBR Green Master Mix (Applied Biosystems) and the ABI PRISM 7900HT sequence detection system (Applied Biosystems) with 10 ng cDNA. The thermal cycling parameters were: step 1, 95°C for 20 s; step 2, 95°C for 1 s; step 3, 60°C for 20 s. Step 2 was repeated for 40 cycles. Relative mRNA expression was calculated from the cycle threshold (Ct) values relative to β-glucuronidase (GUSB) for normalization and the results were expressed as the fold change of control fibroblasts. Primer sequences are listed in Table 1.

**Table 1:** Sequence of primers for RT-PCR.

Gene	Forward primer	Reverse primer
*COX1*	5'-TGGAGCCCCTGATATAGCATTC-3'	5'-CCAGCTTCTACTATGGAGGATGC-3'
*COX2*	5'-GCACAATAGACGCCCAAGAAG-3'	5'-AATTCGTAGGGAGGGAAGGG-3'
*COX4*	5'- TGAACAAGGGCACCAATGA-3'	5'- GCCATACACGTAGCTCTTCTC-3'
*COX5b*	5'-TCCATACAATATGCTACCTCCAAA-3'	5'-ACAGATGCAGCCCACTATTC-3'
*COX10*	5'-TTCCTCAAGCGCATGTATGT-3'	5'-GTGTGCTCAAGAAGAGGAGAAG-3'
*COX17*	5'-AGAAGCCTCTGAAGCCCTGC-3'	5'-GGCCTCGATGAGATGTCCACAG-3'
*TFAM*	5'-GCCTGTCAGCCTTATCTGTATT-3'	5'-TGCATCTGGGTGTTTAGCTTTA-3'
*NRF1*	5'-CTATCCGAAAGAGACAGCAGAC-3'	5'-GGGTGAGATGCAGAGAACAA-3'
*NRF2*	5'-CTGTGATCTGTCCCTGTGTAAA-3'	5'-GGACTTGTGTTCAGCGAAATG-3'
*GUSB*	5'-CAGGAGAGTGGTGTTGAGAATC-3'	5'-TGGTGATGTCAGCCTCAAAG-3'

### Protein determination

Protein concentration was determined by the Lowry method [18].

### Statistics

Statistical significance was assessed by two-tailed student t-test and one-way analysis of variance (ANOVA). A statistical-difference of p<0.05 was considered statistically significant.

## Results

### COX activity and mitochondrial morphology

**Fig 1:**
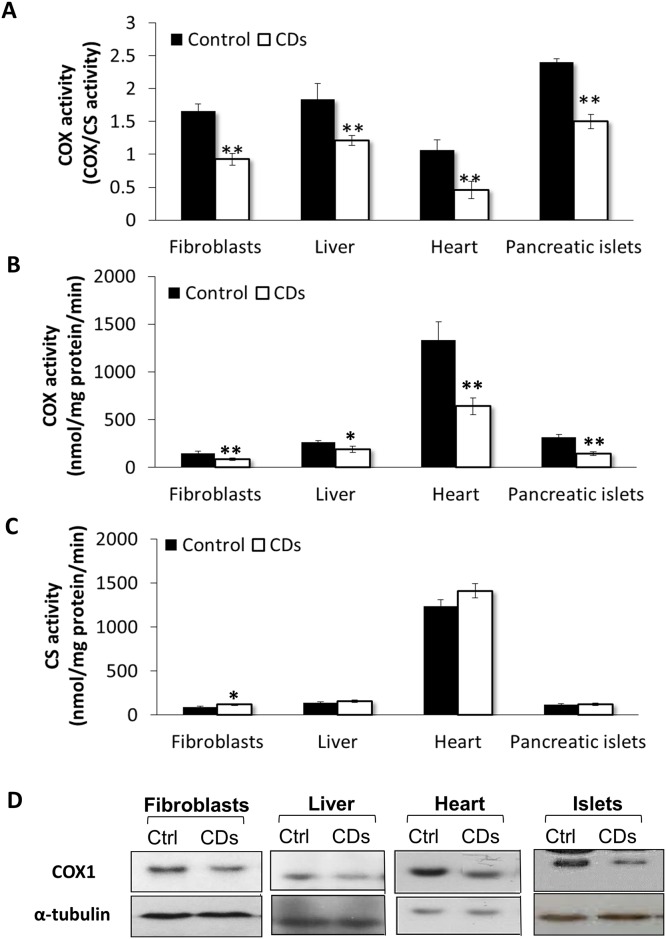
COX deficiency in the CDs rats. (A) COX activity normalized to citrate synthase (CS) activity in fibroblasts, liver, heart and pancreatic-islets of CDs and control rats. (B) COX activity per mg protein. (C) CS activity per mg protein. (D) Western-blot analysis of COX1. Values are mean ± S.E. of three independent experiments. *p<0.05, **p<0.01.

**Fig 2:**
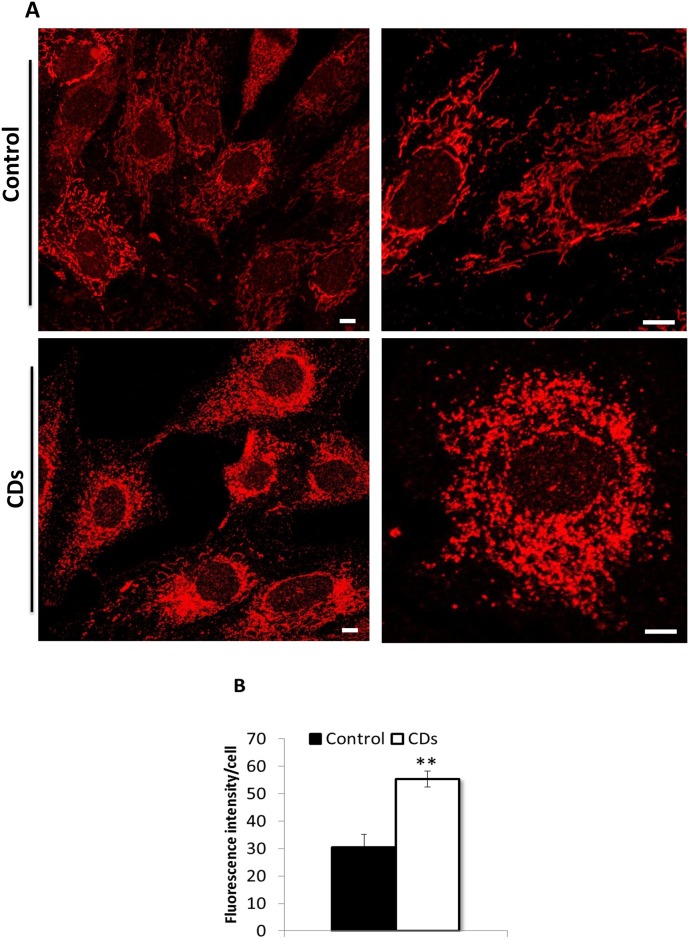
Mitochondrial morphology. (A) Representative confocal microscopy images of control (upper panel) and CDs fibroblasts (lower panel) stained with MitoTracker Red. (B) Quantification of MitoTracker Red fluorescence intensities per cell volume (n = 200 cells). Values are mean ± S.E. of three independent experiments. Scale bar, 10 μm. **p<0.01.

We found decreased COX activity per citrate synthase activity (∼50% of the control) in primary fibroblasts derived from the CDs-rats (Fig 1A; p<0.01). COX to CS activity was also reduced in liver, heart and isolated pancreatic-islets of CDs rats compared with age-matched control rats (Fig 1A; p<0.01), supporting the occurrence of a general COX deficiency in this rat model. COX activity per mg protein was also reduced in all tissues examined from the CDs, while CS activity was mostly unchanged relative to the control (Fig 1B & 1C; p<0.01). In CDs fibroblasts enzymatic activity of CS was slightly but significantly increased (Fig 1C; p<0.05). Moreover, Western blot analysis revealed reduced COX1 subunit protein levels in fibroblasts and tissues of the CDs rats (Fig 1D). To visualize mitochondria fibroblasts were stained with the mitochondria-specific dye MitoTracker Red. In control fibroblasts, mitochondria were organized in extended tubular structures (Fig 2A, upper panel), whereas, in the CDs fibroblasts, the mitochondria morphology was abnormal with rounded and fragmented appearance (Fig 2A, lower panel). The intensity of mitochondrial staining was markedly increased in fibroblasts of the CDs, which may suggest increased mitochondrial mass (Fig 2A, lower panel). Quantification of MitoTracker staining demonstrated an 80% increase in fluorescence intensity in the CDs compared to control fibroblasts confirming the possibility of an increased mitochondrial mass (Fig 2B; p<0.01).

### Cell biomass, mitochondrial content, mitochondrial membrane potential, ATP content and ROS production

**Fig 3:**
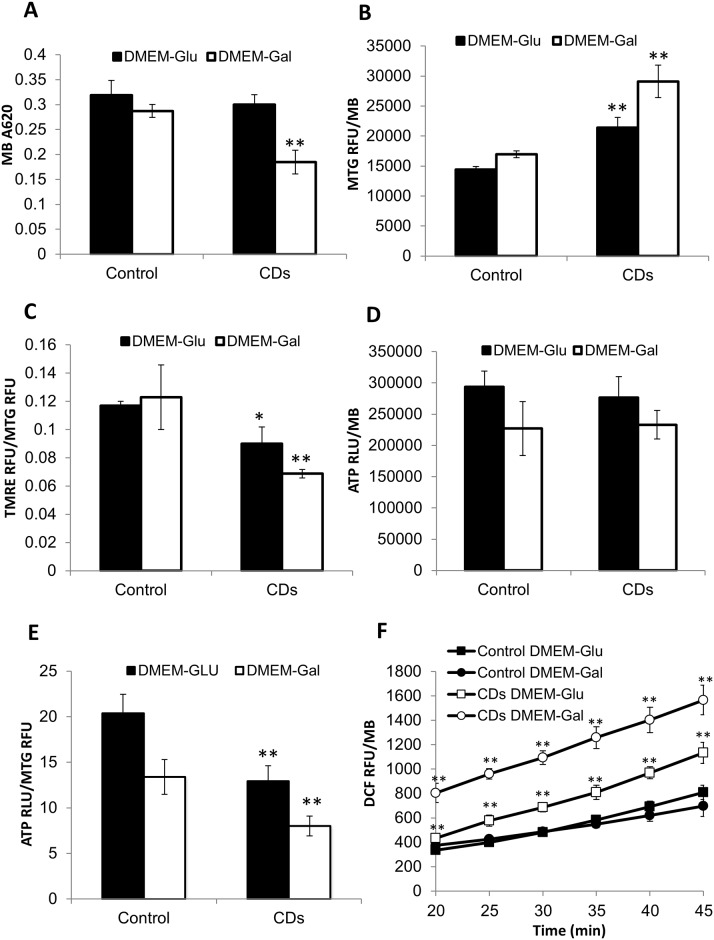
Cell biomass, mitochondrial content, mitochondrial membrane potential, ATP content and ROS production. Fibroblasts were grown in either DMEM-Glu or DMEM-Gal and examined as described under materials and methods. (A) Cell biomass. (B) Mitochondrial content measured by MTG. (C) Mitochondrial membrane potential. (D) Cellular ATP content. (E) ATP content per mitochondria. (F) Kinetics of ROS production. Results are shown as normalized to cell biomass measured by methylene blue (MB) and values are means ± S.E. RFU, relative fluorescence units; RLU, relative luminescence units. (n = 3). *p<0.05, **p<0.01.

The fibroblasts were assessed for ROS production, ATP content, mitochondrial content and membrane potential. Cells were cultured in medium containing glucose that support glycolysis or a medium containing galactose, which does not support glycolysis thus forcing cells to rely on mitochondrial OXPHOS to produce ATP. We found the cell biomass of CDs and control fibroblasts was comparable on permissive DMEM-Glu. However, it was reduced by 35% in the restrictive medium DMEM-Gal that contains galactose as an energy source (Fig 3A; p<0.01 relative to the control fibroblasts). Mitochondrial content was assessed by the mitochondrial dye MitoTracker Green FM that binds mitochondrial membrane independent of the membrane potential, an accepted method to measure mitochondrial mass [19]. In accordance with our previous microscopic observations, we found mitochondrial content in fibroblasts of CDs to be increased by 50% on DMEM-Glu further increasing by 70% on DMEM-Gal, compared with the control (Fig 3B; p<0.01). The mitochondrial membrane potential of CDs-fibroblasts was reduced by 15% on DMEM-Glu and by 45% in and DMEM-Gal, relative to that of the control fibroblasts (Fig 3C; p<0.05). Surprisingly however, the total cellular ATP content of the CDs fibroblasts was comparable to that of the control in both culture media (Fig 3D). Yet, when normalized to mitochondrial content and not to cell biomass, ATP levels were reduced in the CDs fibroblasts (Fig 3E; p<0.01). Kinetic measurement of ROS production showed that the rate was markedly increased in the CDs fibroblasts on both media, but the increase in the DMEM-Gal was much higher (Fig 3F; p<0.01).

### Oxygen Consumption Rate (OCR) and Extracellular Acidification Rate (ECAR)

**Fig 4:**
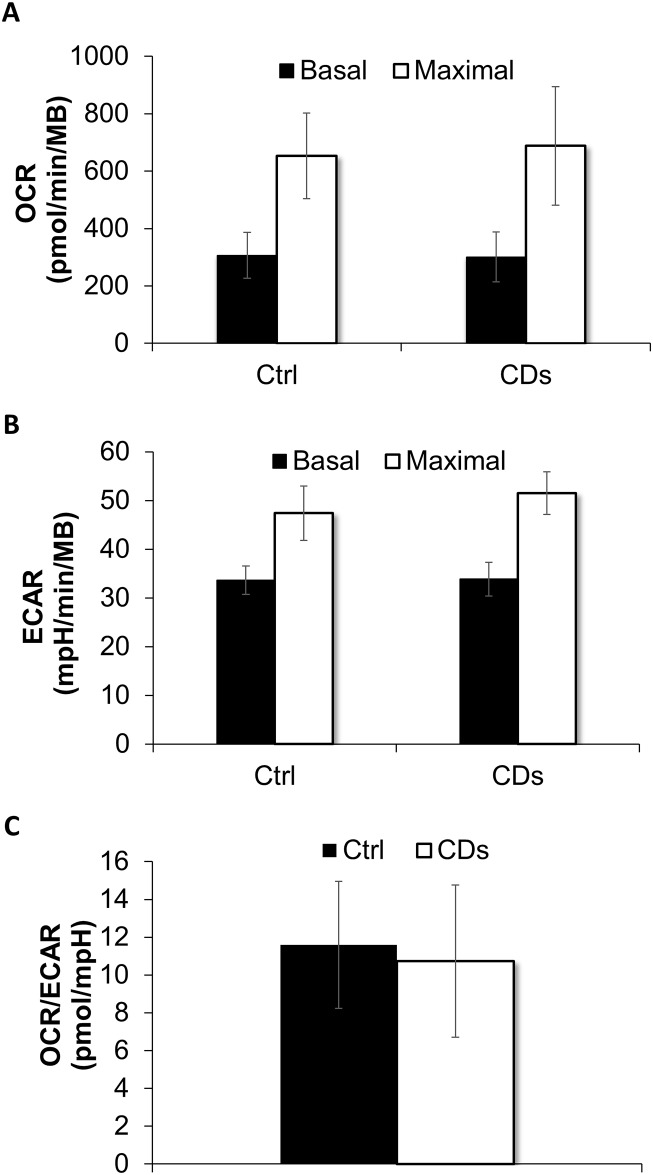
OCR and ECAR. (A) Basal and maximal OCR levels. Basal OCR was measured before Oligomycin injection and maximum OCR after FCCP injection, subtracted by non-mitochondrial respiration (measurement after rotenone and Antimycin A injection). (B) Basal and maximal ECAR levels. Basal ECAR was measured before Oligomycin injection and maximal ECAR levels were measured after the injection of Oligomycin. (C) OCR/ECAR ratio under basal conditions. All OCR and ECAR values were normalized to cell biomass measured by methylene blue (MB). Analysis was performed in five replicate wells for each fibroblast line. Data are mean ± S.E.

We further measured oxygen consumption rate (OCR) and extracellular Acidification Rate (ECAR) which are indicators of mitochondrial respiration and glycolysis respectively. Basal and maximal (stimulated by FCCP) OCR was similar in CDs and the control fibroblasts, suggesting intact mitochondrial respiration (Fig 4A). The basal and maximal (stimulated by Oligomycin) ECAR levels were also comparable in the CDs and control fibroblasts (Fig 4B). Moreover, the OCR/ECAR ratio was similar in the CDs and control fibroblasts, further suggesting no glycolytic shift in metabolism (Fig 4C).

### Mitochondrial biogenesis

**Fig 5:**
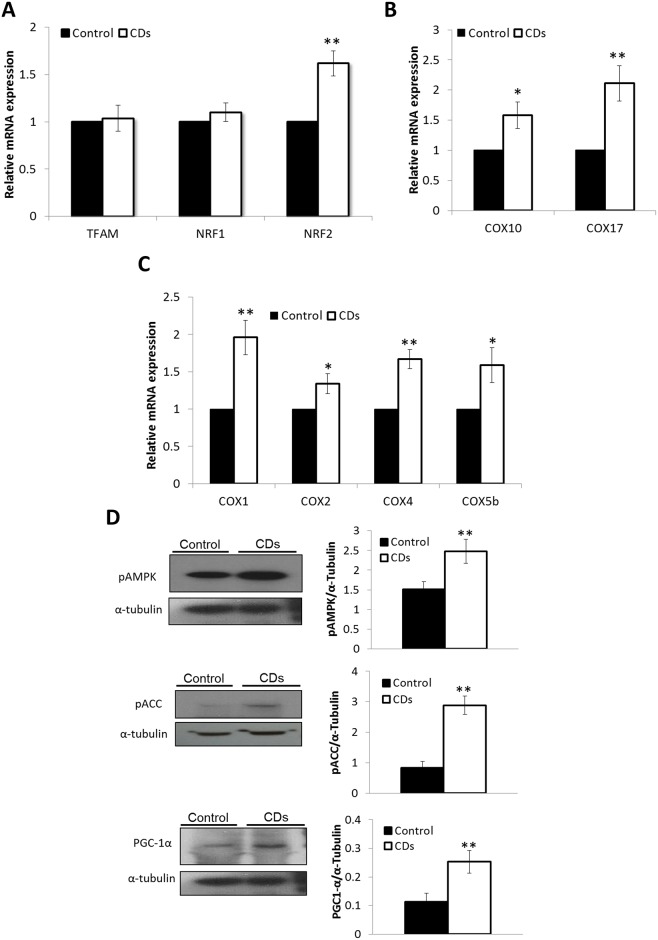
Mitochondrial biogenesis. (A) Relative mRNA-expression of mitochondrial-biogenesis related genes. (B) Relative mRNA-expression of COX subunits. (C) Relative mRNA-expression of COX chaperones. (D) Western-blot analysis of pAMPK, pACC and PGC1-α. On the right panel, band intensity ratios relative to α-Tubulin are shown. Western blots images are representative of 3 independent experiments. Gene expression is shown as fold change of expression relative to control fibroblasts. Data are mean ± S.E. (n = 3). *p<0.05, **p<0.01.

We analyzed the relative mRNA-expression of transcription factors affecting mitochondrial biogenesis TFAM, NRF1 and NRF2 [20]. We found the expression levels of TFAM and NRF1 which mediate mitochondrial biogenesis, was similar in the CDs and the control fibroblasts (Fig 5A). In contrast, the expression of NRF2, that facilitate cellular responses to oxidative stress, was increased by 60% in fibroblasts of CDs, supporting the occurrence of oxidative stress (Fig 5A; p<0.01). Although the mRNA-expression of NRF1 and TFAM was unchanged, several of their downstream targets including COX subunits COX1, COX2, COX4 and COX5b as well as COX chaperones COX10 and COX17 were all upregulated in the CDs fibroblasts relative to the control (Fig 5B & 5C; p<0.05), possibly indicating increased synthesis. To gain further insight into the possible mechanism underlying mitochondrial biogenesis in CDs we measured AMPK phosphorylation and activity, evident by the phosphorylation of its downstream target acetyl-CoA carboxylase (ACC) by western-blot. We observed increased pAMPK levels in the CDs fibroblasts compared with the control fibroblasts (Fig 5E; p<0.01). In agreement, levels of pACC were significantly higher in the CDs fibroblasts (Fig 5E; p<0.01). In addition, levels of PGC-1α, a transcriptional regulator for genes involved in mitochondrial biogenesis, were also increased in the CDs fibroblasts (Fig 5E; p<0.01).

### Mitophagy

**Fig 6:**
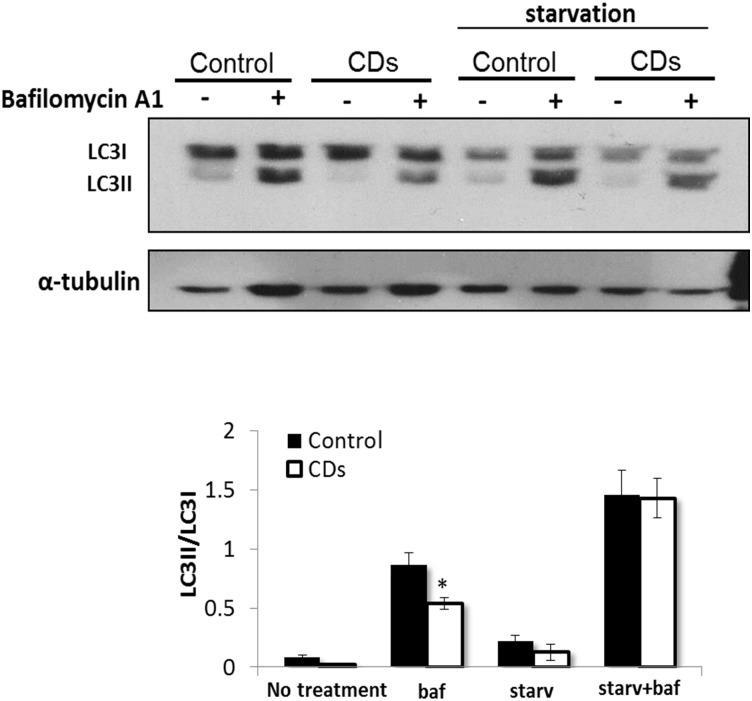
Autophagy analysis. Western-blot analysis of LC3-I and LC3-II. Fibroblasts were grown under normal or starvation (starv) conditions in the presence or absence of autophagy inhibitor bafilomycin A1 (baf, 100 nM) for 4 h as described under materials and methods. Lower panel shows the band intensity ratio of LC3-II to LC3-I. Data represent mean ± S.E. of three independent experiments. *p<0.05.

**Fig 7:**
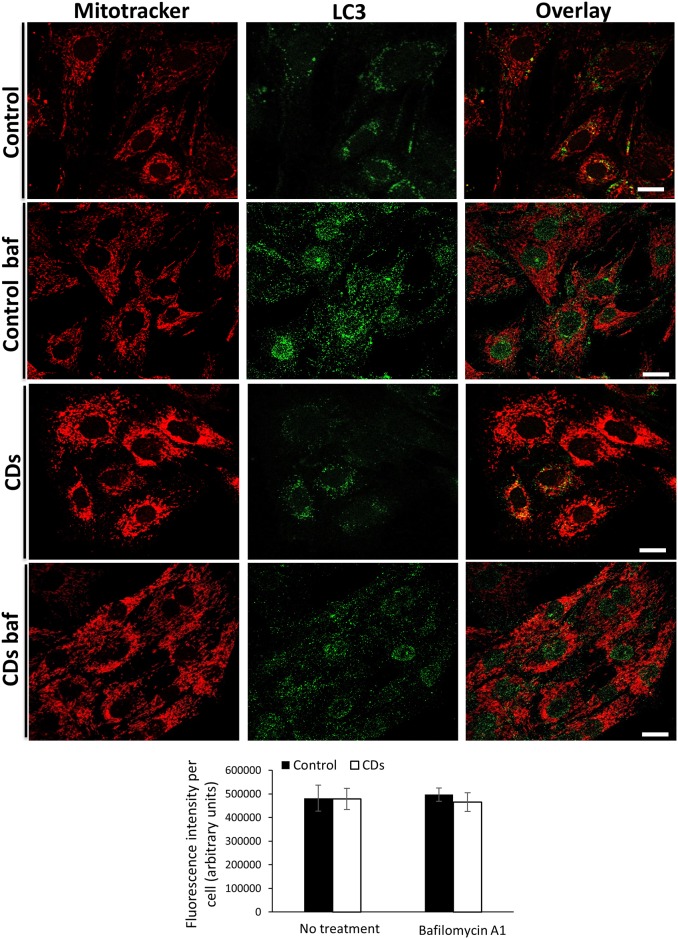
Mitophagy analysis. Co-staining with Mitotracker Red and anti-LC3 antibody in the presence or absence of autophagy inhibitor Bafilomycin A1. The upper panels shows the control fibroblasts and the lower panels the CDs fibroblasts. Below is shown quantitative analysis of fluorescence intensity (arbitrary units) of LC3 and MitoTracker Red co-localization per cell. Data represent mean ± S.E. of three independent experiments. Scale bar, 10 μm.

We sought to examine the possibility that the increase in mitochondrial mass observed in the CDs fibroblasts is due to decreased mitochondrial clearance by autophagy. To study autophagy we assessed endogenous LC3-I and LC3-II by western-blotting. During autophagosome formation, the LC3-I isoform is converted into LC3-II, thus serving as a marker of autophagosomes number and the induction of autophagy [21,22]. When grown either in a complete media or under starvation conditions a comparable low LC3-II levels and LC3-II/LC3-I ratio could be detected in the CDs and the control fibroblasts (Fig 6). Autophagy inhibition by bafilomycin A1, that inhibits autolysosomal degradation [23], induced a significant and similar increase in LC3-II levels and the LC3-II/LC3-I ratio in the CDs and control fibroblasts, under both normal and starvation conditions (Fig 6), indicating normal autophagic flux. Since some mitochondrial defects increase specific degradation of mitochondria (mitophagy) [24], we analyzed the co-localization of Mitotracker Red with LC3, as an index of mitophagy, by confocal microscopy [25,26]. We observed LC3-positive structures in both the CDs and the control fibroblasts, with no apparent differences (Fig 7A). Moreover, LC3 co-localization with the mitochondria with or without Bafilomycin A1 was similar in the CDs and the control fibroblasts (Fig 7A). Further quantification of fluorescence intensity of LC3 signal co-localized with mitochondria (MitoTracker Red) showed no difference in co-localization per cell (Fig 7B), indicating no major autophagic mitochondrial sequestration.

## Discussion

Here we demonstrate a decreased COX activity associated with fragmented mitochondria, enhanced ROS production and reduced mitochondrial membrane potential in primary fibroblasts derived from CDs rats. Interestingly, mitochondrial content was upregulated as were markers for mitochondrial biogenesis, probably representing an attempt to compensate for COX-deficiency, thus providing a possible explanation for the unexpectedly intact ATP levels in fibroblasts of the CDs.

The increase in mitochondrial mass observed in the CDs fibroblasts could be due to increased mitochondrial biogenesis, decreased mitochondrial clearance by autophagy or both. Increased mitochondrial biogenesis is a common observation in tissues of patients with mitochondrial dysfunction and is assumed to be a compensatory mechanism triggered by the OXPHOS defect [27–29]. However, with regard to COX-deficiency, this has been hardly documented. In the current study, we demonstrated a 50% reduction in COX activity in the CDs fibroblasts, followed by a 2-fold increase in mitochondrial content. We hypothesized that the increase in mitochondrial content in cells of the CDs is the result of increased mitochondrial biogenesis that might be mediated through activation of AMPK, a major regulator of mitochondrial biogenesis, triggered by phosphorylation at threonine-172 [30,31]. Upon activation, phosphorylated AMPK (pAMPK) acts simultaneously on boosting mitochondrial energy production and inhibiting ATP consuming processes [32]. Using Western-blot analysis, we demonstrated elevated pAMPK levels in fibroblasts of the CDs. Accordingly, the protein levels of pAMPK downstream key-targets, PGC-1α and pACC were also markedly increased, supporting AMPK activation. Our findings are consistent with studies showing elevated pAMPK levels in skeletal-muscle of COX-defective mouse models [33]. The relative expression levels of the transcription factor NRF1, which promotes mitochondrial function and biogenesis [20] were unchanged, whereas the expression of several NRF1 immediate target genes including COX1, COX2, COX4, COX5b, COX10 and COX17 were all upregulated in fibroblasts of the CDs. These findings in the CDs are fully compatible with the role of PGC-1α as a transcription coactivator increasing the transcriptional activity of NRF1. To test if the observed enhanced mitochondrial staining in the CDs fibroblasts could be due to mitochondrial accumulation caused by aberrant autophagy clearance, we monitored the endogenous levels of autophagy marker LC3-II [21]. The observation that only low LC3-II levels were found in CDs fibroblasts grown under normal conditions suggested that basal autophagy was not elevated. Autophagy was efficiently induced in starved CDs fibroblasts as demonstrated by the comparable increase in both LC3-II levels and the LC3-II/LC3-I ratio in CDs and control fibroblasts. Moreover, the similar increase in LC3-II levels and the LC3-II/LC3-I ratio following a specific inhibition of autophagy by bafilomycin A1 in both CDs and the control fibroblasts, further support normal autophagic-flux. To reinforce our findings we examined whether the mitochondrial fragments in cells of CDs were associated with LC3 but could not detect any difference in LC3 subcellular localization to the mitochondria between the CDs and the control fibroblasts. These observations might indicate no major autophagy impairment or enhanced autophagic mitochondrial elimination in the CDs fibroblasts, supporting the notion that upregulation of mitochondrial content in the CDs might be due to mitochondrial biogenesis activation. This could reflect cellular attempts to compensate for COX-deficiency in order to prevent energy stress, possibly mediated by the AMPK pathway.

Fibroblasts in culture generate most of their ATP via glycolysis and not by OXPHOS [34,35]. The DMEM-Gal, containing galactose instead of glucose as a carbon source, does not support glycolysis, thus forcing cells to use the mitochondrial OXPHOS to produce ATP. Thereby, fibroblasts having OXPHOS defects are highly sensitive to the DMEM-Gal [36–38]. We have shown that the CDs fibroblasts exhibited reduced cell biomass on DMEM-Gal compared to the control fibroblasts and to the CDs glucose-grown cells, suggesting they primarily rely on glycolysis for energy production. In agreement with the microscopic findings showing increased mitochondrial-staining in the CDs-fibroblasts, quantitative measurement of MitoTracker Green stained cells, a dye that binds to mitochondria regardless of their membrane potential, also demonstrated increased mitochondrial content in the CDs-fibroblasts on both the glucose and galactose medium. Moreover, both cellular OCR, resulting from oxidative phosphorylation, and ECAR, associated with glycolytic metabolism as well as the OCR/ECAR ratio were intact in the CDs fibroblasts. These results indicates that the COX defect was not sufficient to reduce oxygen consumption, likely due to the increase in mitochondrial mass, and does not support the occurrence of a shift from OXPHOS towards a glycolytic state. However, mitochondrial membrane potential and the ATP levels per mitochondria in CDs-fibroblasts were decreased. Yet, the cellular ATP levels in the CDs fibroblasts were unchanged in either the glucose or galactose media. We propose that these conflicting results could be the outcome of an adaptive cellular response to the metabolic environment attempting to overcome COX defect by increasing mitochondrial biomass. Following our assumption, the CDs fibroblast maintained unchanged total cellular-ATP level though ATP levels per mitochondria were reduced, as a consequence of the increase in mitochondria content triggered by reduction in COX activity and in mitochondrial membrane potential. Yet, this compensatory mechanism was not observed in islets and could be specific to the fibroblasts [12].

Mitochondria are one of the major cellular sources of ROS and mitochondrial dysfunction often leads to ROS overproduction. Indeed, in the current study, fibroblasts of the CDs exhibited increased ROS production on DMEM-Glu, enhancing remarkably on DMEM-Gal, possibly due to COX-dysfunction and the increased mitochondrial mass. Our findings are consistent with previous observations showing elevated ROS levels in COX-deficient fibroblasts [39]. Supporting an increase in oxidative stress, we found the expression of the transcription factor NRF2, that facilitate cellular antioxidant response to oxidative stress [40] to be upregulated in fibroblasts of CDs rats. The cellular role of mitochondria is reflected by their structure. In accordance, the CDs-fibroblasts displayed altered mitochondrial morphology including pronounced fragmentation and perinuclear clustering compared to the elongated filamentous mitochondria observed in the control fibroblasts. Similarly, others have also shown that treating cells with COX inhibitor KCN induced mitochondrial fragmentation [41]. Punctate mitochondrial appearance was also observed in samples from respiratory complex I and multi-complex deficient patients [34] and several studies showed that inhibition of mitochondrial respiratory complexes I–V induced mitochondrial fragmentation in human skin fibroblasts [41–44]. These may suggest a crosstalk between mitochondrial dysfunction and mitochondrial morphology in which changes in mitochondrial morphology could reflect alterations in energy metabolism.

Our data suggest that COX dysfunction in the CDs fibroblasts triggers upregulation of mitochondrial content due to the stimulation of mitochondrial biogenesis rather than impaired autophagic clearance. This is likely to occur by activation of the AMPK-dependent pathway, thereby counteracting the detrimental effect of COX-deficiency thus allowing the CDs fibroblasts to maintain overall ATP levels unaffected.
